# Efficient production of polymer-grade L-lactic acid from corn stover hydrolyzate by thermophilic *Bacillus* sp. strain XZL4

**DOI:** 10.1186/2193-1801-1-43

**Published:** 2012-10-29

**Authors:** Zhangwei Xue, Limin Wang, Jiansong Ju, Bo Yu, Ping Xu, Yanhe Ma

**Affiliations:** 1 Institute of Microbiology, Chinese Academy of Sciences, Beijing, 100101 China; 2 State Key Laboratory of Microbial Metabolism and School of Life Sciences and Biotechnology, Shanghai Jiao Tong University, Shanghai, 200240 China; 3 College of Life Science, Hebei Normal University, Shijiazhuang, 050016 China

**Keywords:** L-lactic acid, Thermophilic, *Bacillus* sp, Corn stover hydrolyzate

## Abstract

Lactic acid has been identified as one of the top 30 potential building-block chemicals from biomass. Therefore, the search for cheap raw materials is an objective to reduce the production costs. Efficient polymer-grade L-lactic acid production was achieved in this report by a thermophilic strain *Bacillus* sp. XZL4 using corn stover hydrolyzate as sole carbon source. High L-lactic acid concentration (81.0 g L^-1^) was obtained from 162.5 g L^-1^ concentrated corn stover hydrolyzate (total reducing sugar of 83.0 g L^-1^) with a volumetric productivity of 1.86 g L^-1^ h^-1^ (0–36 h) and a product yield of 0.98 g g^-1^ total reducing sugars. This is the highest L-lactic acid concentration and yield reported from corn stover hydrolyzate. And the high optical purity of L-lactic acid obtained in this study also indicated that *Bacillus* sp. XZL4 is a promising polymer-grade L-lactic-acid producer from cellulosic biomass.

## Background

Lactic acid is a valuable chemical and one of its extensive applications is for polymerization of L-lactic acid to poly (L-lactic acid), which is an attractive polymer because it can be produced from renewable resources and is biodegradable. These properties have strengthened interest in developing more efficient production processes for optical purity of L-lactic acid (Wang et al. 2010a). As lactic acid has been identified as one of the top 30 potential building-block chemicals from biomass (http://www.eere.energy.gov/biomass/pdfs/35523.pdf), the search for cheap raw materials is some of the most important objectives to be achieved to reduce the costs. To improve productivity and economy of lactic acid production, some reports have investigated the potential of utilizing low-cost raw materials as carbon sources, such as molasses and cellulosic materials (Patel et al. 2004; Romaní et al. 2008; Wang et al. 2010a). Corn stover, one of the lignocellulosic biomasses, is the agricultural residue left unutilized in harvested. Corn stover is not a food source and has high concentration of mixed sugars, mainly including glucose and xylose, and therefore it is considered as one of the most important global feedstocks for the production of chemicals in future (Georgieva and Ahring 2007). Furthermore, the addition of large amounts of yeast extract in lactic acid fermentation was also economically prohibited for producing such low-value biocommodities (Altaf et al. 2007). Various low-cost raw materials such as tryptic soy (Nancib et al. 2005), soybean hydrolyzate (Kwon et al. 2000), corn steep liquor (Nancib et al. 2001), whey protein hydrolyzate (Fitzpatrick and O’Keeffe 2001) and red lentil and baker’s yeast cells (Altaf et al. 2006) have been investigated to substitute yeast extract for lactic acid production. However, most of the substitutes were not very effective. Therefore, development of an efficient and cost-effective process for lactic acid fermentation from cheap and non-food substrates is highly desired.

*Bacillus* species are the most widely utilized microorganisms for L-lactic acid production (Budhavaram and Fan 2009; Danner et al. 1998; Patel et al. 2004Qin et al. 2010; Walton et al. 2010; Wang et al. 2010b; Zhao et al. 2010). As potential industrial strains, thermophilic *Bacillus* species offers several remarkable advantages for lactic acid production, including the reduction of contamination from competing microbes, simple nutrition requirements, and simple maintenance of stock cultures (Patel et al. 2004; Qin et al. 2009). In this study, corn stover hydrolyzate was chosen as sole carbon and different low-cost nitrogen sources (peanut meal, dry corn syrup and soybean meal) were tested as sole nitrogen source, respectively, for polyer-grade L-lactic acid production by a thermophilic *Bacillus* strain. The aim of this study was to develop an encouraging process for the economical L-lactic acid production based on cheap raw materials. The batch fermentation results of high yield and high optical purity of L-lactic acid from corn stover hydrolyzate indicated that *Bacillus* sp. strain XZL4 used in this study is a promising L-lactic-acid producer from cellulosic biomass.

## Results

### Effects of corn stover hydrolyzate concentrations on L-lactic acid production

**Figure 1:**
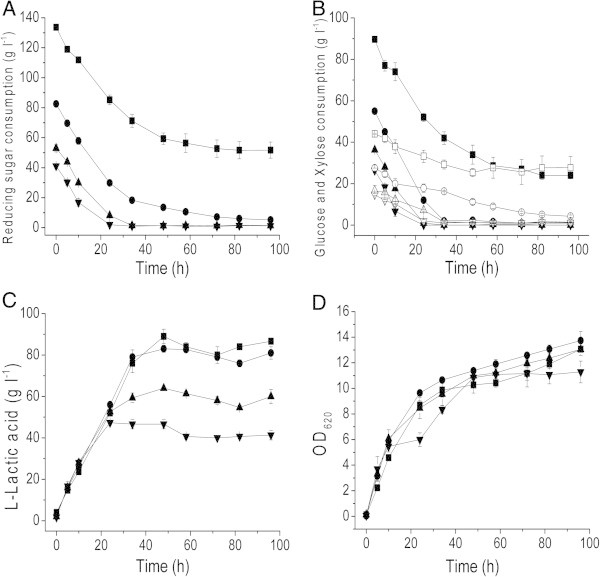
**Effects of corn stover hydrolyzate concentrations on L-lactic acid production by*Bacillus*sp. XZL4.** (**A**) The total reducing sugars consumption. (**B**) Glucose and xylose consumption. (**C**) L-lactic acid production. (**D**) Cell growth. Symbols represent different initial concentrations of total reducing sugars of corn stover hydrolysis (g L^-1^): 133 (■), 83 (●), 53 (▲), and 41 (▼). The symbols for xylose consumption under different initial concentrations of total reducing sugars of corn stover hydrolysis (g L^-1^) were 133 (□), 83 (○), 53 (△), and 41 (▽), respectively. The error bars in the figure indicate the standard deviations of three parallel replicates.

Different concentrations of corn stover hydrolyzate with the total reducing sugars of 41, 53, 83 and 133 g L^-1^, respectively were firstly used to test the effects of corn stover hydrolyzate concentrations on L-lactic acid production. As shown in Figure 1, when the initial corn stover hydrolyzate concentration was below 162.5 g L^-1^ (total reducing sugar is 83 g L^-1^), L-lactic acid concentration increased with the addition of corn stover hydrolyzate. Glucose in corn stover hydrolyzate was depleted within 48 h, and after 72 h of incubation, the total reducing sugars were almost completely consumed (Figure 1). It is notable that *Bacillus* sp. strain XZL4 could utilize both the two sugars simultaneously, although glucose was utilized a little faster than xylose. When the initial reducing sugar concentration condition reached 133.7 g L^-1^, strain XZL4 could only consume approximately 80 g L^-1^ reducing sugars and produce almost the same amount of lactic acid as the culture with 83 g L^-1^ initial total reducing sugars. Further increasing the reducing sugar concentration could not lead to the increase of lactic acid concentration, so 83 g L^-1^ initial total reducing sugar concentration was chosen for the subsequent studies.

### Effects of nitrogen source on L-lactic acid production by strain XZL4

**Figure 2:**
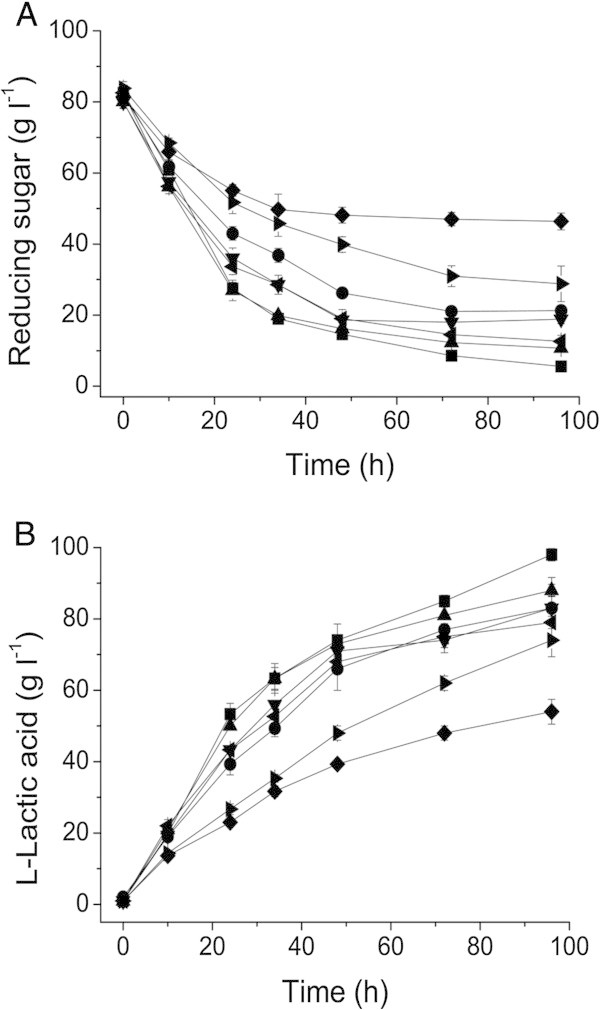
**Effects of different nitrogen sources on L-lactic acid production by*Bacillus*sp. XZL4.** (**A**) Reducing sugars consumption. (**B**) L-lactic acid production. Symbols represent different nitrogen sources in the fermentation medium: yeast extract (■), dry corn syrup (●), peanut meal (▲), soybean meal (▼), peptone (◂), (NH_4_)_2_SO_4_ (▸), and (NH_4_)_2_HPO_4_ (◆). The error bars in the figure indicate the standard deviations of three parallel replicates.

To reduce the cost, different kinds of organic and inorganic nitrogen sources were investigated to substitute the expensive yeast extract during L-lactic acid fermentation. Quantities of the nitrogen sources used for L-lactic acid production were added corresponding to a nitrogen concentration of 4.5 g L^-1^. As shown in Figure 2, when strain XZL4 was cultivated in a medium containing two kinds of the inorganic nitrogen source ((NH_4_)_2_SO_4_ and (NH_4_)_2_HPO_4_), few L-lactic acid was produced. In the medium with 12 g L^-1^ dry corn syrup and 6 g L^-1^ soybean meal, the produced L-lactic acid concentrations were 77 g L^-1^ and 78 g L^-1^, respectively. A higher L-lactic acid concentration was obtained with peanut meal as nitrogen source although it was a little lower than that of yeast extract (Figure 2).

**Figure 3:**
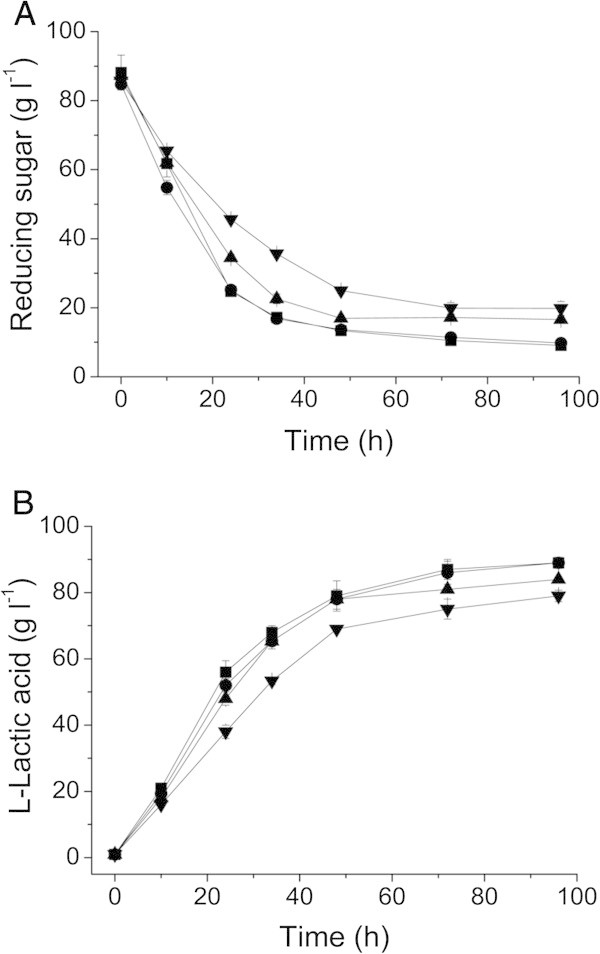
**Effects of different nitrogen concentrations on L-lactic acid production by*Bacillus*sp. XZL4.** (**A**) Reducing sugars consumption. (**B**) L-lactic acid production. Symbols represent different concentration of peanut meal in the fermentation medium (g L^-1^): 13.2 (■), 9.9 (●), 6.6 (▲), and 3.3 (▼). The error bars in the figure indicate the standard deviations of three parallel replicates.

To investigate the effects of peanut meal concentrations on L-lactic acid production, strain XZL4 was cultivated in fermentation medium with different initial concentrations of peanut meal (3.2, 6.6, 9.9 or 13.2 g L^-1^). Figure 3 showed that L-lactic acid concentration increased with the addition of peanut meal and 9.9 g L^-1^ peanut meal could meet the requirement for L-lactic acid production.

### L-Lactic acid production from corn stover hydrolyzate by batch fermentation

**Figure 4:**
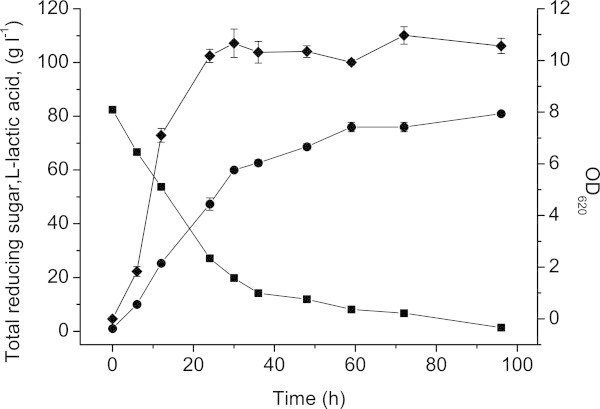
**Batch fermentations from corn stover hydrolyzate by*Bacillus*sp. XZL4.** Symbols represent carbohydrates consumption and cell growth in the fermentation medium (g L^-1^): Reducing sugars consumption (■), L-lactic acid production (●), and Cell growth (◆). The error bars in the figure indicate the standard deviations of three parallel replicates.

Batch fermentations were performed in a 3-L Erlenmeyer flask containing 1 L fresh medium, with initial concentration of 162.5 g L^-1^ corn stover hydrolyzate (83 g L^-1^ of total reducing sugars). The lactic acid concentration reached 63 g L^-1^ within 36 h and the average L-lactic acid productivities of this time period were 1.86 g L^-1^ h^-1^ (Figure 4). The production of lactic acid terminated at 96 h with a final concentration of 81.0 g L^-1^ with only trace amounts of acetic acid detected (< 0.1 g L^-1^), when the residual reducing sugars were almost completely consumed. The yield of lactic acid was 0.98 g g^-1^ total reducing sugars, which was very close to its theoretical value. No D-isomer of lactic acid was detected in the broth.

## Discussion

Inexpensive underutilized agricultural by-products, such as corn stover hydrolyzate offers an attractive possibility to be used as substrate in biotechnological production of L-lactic acid, but the lactic acid yield and volumetric productivity in fermentation of agricultural by-products are generally low. The lactic acid production titers from different agricultural by-products were summarized in Table 1. Relatively low lactic acid concentrations were obtained when lime-treated wheat straw hydrolyzate (Maas et al. 2008), cellobiose (Abdel-Rahman et al. 2011), sugar cane bagasse hemicellulose hydrolyzate (Patel et al. 2004), and corn fiber hydrolyzate (Walton et al. 2010) were used for lactic acid production. *Lactobacillus pentosus* ATCC 8041 has been reported to produce 74.8 g L^-1^ lactic acid from corn stover hydrolyzate and yeast extract by fed-batch fermentation process while the low productivity (0.26 g L^-1^ h^-1^) and yield (0.65 g g^-1^) were reported (Zhu et al. 2007a). To further reduce the cost, different kinds of organic and inorganic nitrogen sources were also investigated to substitute the expensive yeast extract during L-lactic acid fermentation in this study. Peanut meal is the high-protein solid residue gained from the extraction of peanut oil, which is cheap and very abundant in China. Substitution of yeast extract with peanut meal will significantly reduce the production cost of lactic acid. High concentration of D-lactic acid was obtained using peanut meal as nitrogen source by *Sporolactobacillus* sp. CASD (Wang et al. 2011). *Bacillus* sp. strain XZL4 could efficiently produce L-lactic acid from corn stover hydrolyzate with a yield of 0.98 g g^-1^ reducing sugars using peanut meal as sole nitrogen source. Our results demonstrated that peanut meal was an efficient and economic nitrogen alternative for polymer-grade L-lactic acid fermentation by thermophilic *Bacillus* strains.

**Table 1:** **Comparison of lactic acid production from agricultural by-products by lactic acid producing microorganisms**

			Lactic acid			
Substrate	Organism	Fermentation process	Lactic acid concentration (g/L)	Productivity (g/L/h)	Yield (g/g)	References
lime-treated wheat straw hydrolyzate	*Bacillus coagulans* DSM 2314	Continuation of the SSF	40.7^a^	0.74	0.43	Maas et al. [2008]
sugar cane bagasse hemicellulose hydrolyzate	thermotolerant acidophilic *Bacillus* sp. strain 17C5	Batch	55.8^a^	0.8	0.93	Patel et al. [2004]
cellobiose	*Enterococcus mundtii* QU25	Batch	20.4^a^	3.44	1.04	Abdel-Rahman et al. [2011]
reed hemicellulose liquor	*Lactococcus lactis* IO-1 JCM 7638/ *Lactobacillus pentosus* ATCC 8041	Batch	33.0^c^	0.6	0.66	Perttunen et al. [2001]
wheat straw hydrolyzate	fungus *Rhizopus oryzae* CBS 112.07	Batch	6.8^a^	0.14	0.23	Maas et al. [2006]
hot water-extracted Siberian larch	moderate thermophile *Bacillus coagulans* MXL-9	SSF	33.0^a^	0.55	0.73	Walton et al. [2010]
paper sludge	*Bacillus coagulan* strains 36D1	SSCF	92.0^c^	0.96	0.77	[Budhavaram and Fan 2009]
defatted rice bran	*Lactobacillus delbrueckii* IFO 3202	SSF	28.0^b^	0.78	0.28	Tanaka et al. [2006]
wood hydrolyzate	*Enterococcus faecalis* RKY1	Batch	93.0^a^	1.7	0.93	Wee et al. [2004]
corn fiber hydrolyzate	moderate thermophile *Bacillus coagulans* MXL-9	Fed batch	45.6^a^	0.21	0.46	Bischoff et al. [2010]
corn stover hydrolyzate	*Lactobacillus pentosus* ATCC 8041	Fed batch	74.8^c^	0.26	0.65	Zhu et al. [2007a]
corn stover hydrolyzate	*Bacillus* sp. strain XZL4	Batch	81.0^a^	1.86(0–36 h)	0.98	This work

SSF: Simultaneous saccharification and fermentation.

SSCF: Semi-continuous simultaneous saccharification and co-fermentation.

^a^ L-lactic acid.

^b^ D-lactic acid.

^c^ DL-lactic acid.

Additionally, cellulose- and hemicellulose-derived carbohydrate feedstocks contained a variety of mixed sugars, mainly glucose and xylose. In order to maximize lactic acid yield and production, complete utilization of mixed sugars is essential. Carbon catabolite repression (CCR) is a common phenomenon in bacteria and very few bacteria have been reported which consume different sugars simultaneously (Görke and Stülke 2008). Therefore, for industrialization of lactic acid production from cellulosic materials, it is desirable to use CCR-positive strain for lactic acid production from mixed sugar substrates. *Bacillus* sp. strain XZL4 could utilize both the two sugars simultaneously, although glucose was utilized a little faster than xylose (Figure 1), proving its feasibility for L-lactic acid production from low-cost raw materials.

High L-lactic acid concentration with a volumetric productivity of 1.86 g L^-1^ h^-1^ (0–36 h) and a product yield of 0.98 g g^-1^ total reducing sugars was obtained in batch fermentation by *Bacillus* sp. strain XZL4. The mechanisms that strain XZL4 produced L-lactic acid so effectively could be explained by the analysis results from genome sequencing data (Su et al. 2011). The genome size of *Bacillus* sp. strain XZL4 is only 2.8 Mb. The small genome size with less genomic redundancy was thought to improve the productivity of platform chemicals or other products (Zhu et al. 2007b; Morimoto et al. 2008). The pathway of EMP is well known for its high efficiency to utilize hexose. Compared to the hexose, the pathways of utilization of pentose are more flexible. In theory, the transketolase/transaldolase pathway has higher carbon efficiency than the phosphoketolase pathway. Based on carbohydrate metabolism analysis, the key enzymes (xylose/arabinose isomerase, ribulokinase, and ribulose-5-phosphate 4-epimerase) involved in the pentose metabolite were found in the genome. The transketolase/transaldolase pathway, instead of phosphoketolase, was in the genome, implying that strain XZL4 could utilize pentose more efficiently. Furthermore, few pyruvate-dissipating enzymes were found in strain XZL4 (Su et al. 2011). Therefore, simple and efficient carbohydrate metabolism systems, especially the absence of pyruvate decarboxylase and the existed transketolase/transaldolase pathway in thermophilic *Bacillus* sp. strain XZL4, should be responsible for the high-yield lactic acid production from corn stover hydrolyzate. Additionally, the absence of D-lactate dehydrogenase genes in the genome of *Bacillus* sp. strain XZL4 also resulted in producing such high-optical purity L-lactic acid.

## Conclusions

In conclusion, the highest lactic acid concentration (81.0 g L^-1^) and yield (0.98 g g^-1^ total reducing sugars) was obtained from corn stover hydrolyzate in this study. Corn stover hydrolyzate can provide an economic L-lactic acid production process with cheap and renewable biomass by *Bacillus* sp. strain XZL4. Although the relatively higher concentrations of lactic acid were previously reported by using paper sludge (Budhavaram and Fan 2009) and wood hydrolysate (Wee et al. 2004) (Table 1), expensive yeast extract used as nitrogen source reduced their industrial applicability. And more, the absence of pyruvate decarboxylase and D-lactate hydrogenase genes in the genome demonstrated that *Bacillus* sp. strain XZL4 is a high-efficient polymer-grade L-lactic-acid producer from cellulosic biomass.

## Methods

### Chemicals

The corn stover hydrolyzate, kindly provided by Energy Research Institute of Shandong Academy of Sciences (China), was prepared by following the procedures: 1) Pretreatment: The corn straw was grinded, then the powder was sieved and the particle sizes ≤2 mm were collected; 2) HCl treatment: The collected powder was treated with 6% HCl at 90°C for 1 h, then washed by water and adjusted to pH 4.8-5.0; 3) Cellulase hydrolysation: The powder of corn straw was hydrolyzed with cellulase (20 FPIU/g dry mass, solid–liquid ratio was 1:50–1:10) at 50°C for 48 h, and the corn straw hydrolysate was concentrated and used in this study. The contents of concentrated corn stover hydrolyzate was glucose 555.3 (g L^-1^), xylose 174.2 (g L^-1^), arabinose 19.9 (g L^-1^), acetic acid 7.1 (g L^-1^), 2-furfural 1.9 (g L^-1^) and 5-hydroxymethyl-2-furaldehyde 0.7 (g L^-1^). All other chemicals were of analytical grade and commercially available.

### Strain and culture conditions

*Bacillus* sp. strain XZL4 used in this study is a homofermentative L-lactic acid producer (Su et al. 2011). The strain has been deposited in the Deutsche Sammlung von Mikroorganismen und Zellkulturen GmbH (DSM №23183). The slant was inoculated at 50°C after 24 h of incubation and stored at 4°C. Strain XZL4 was inoculated in the culture medium containing (per liter) 50 g glucose, 10 g yeast extract (YE), 30 g calcium carbonate (Wang et al. 2010b). The seed culture was prepared as follows: a loop of cells from the fully grown LB agar slant was inoculated into 30 mL of the above sterile medium in 100-mL conical flasks and incubated at 50°C for 24 h without agitation. All experiments were 10% (v/v) inoculum volume and carried out in triplicate.

### Effects of corn stover hydrolyzate concentrations on L-lactic acid production

The medium used to study the effects of corn stover hydrolyze concentrations on L-lactic acid production contained 41–133 g L^-1^ reducing sugars and 10 g L^-1^ YE. Calcium carbonate was added as 60% (w/w) of the reducing sugars to the medium (Wang et al. 2010a). The well mixed samples were taken periodically and the concentrations of total residual reducing sugar, glucose, xylose and the L-lactic acid production were determined.

### Effects of different nitrogen sources on L-lactic acid production

The variables used in the study were peanut meal and soybean meal (with 0.3 g L^-1^ neutral proteinase, respectively, to release the nitrogen element), peptone, yeast extract, dry corn syrup, (NH_4_)_2_SO_4_ and (NH_4_)_2_HPO_4_. The 162.5 g L^-1^ corn stover hydrolyzate (containing 83.0 g L^-1^ reducing sugar) and 49.5 g L^-1^ CaCO_3_ were added. The quantities of nitrogen in all medium were controlled at 4.5 g L^-1^. The well mixed samples were taken periodically and the concentrations of total residual reducing sugar and the L-lactic acid production were determined.

### Effects of peanut meal concentrations on L-lactic acid production

The fermentation medium for studying nitrogen concentration utilization contained 1.3-13.2 g L^-1^ peanut meal and 162.5 g L^-1^ corn stover hydrolyzate, calcium carbonate was added as 60% (w/w) of reducing sugars to the medium. Fermentations were carried out at 50°C under static conditions in 100-mL Erlenmeyer flasks each containing 30 mL medium. The well mixed samples were taken periodically and the concentrations of total residual reducing sugar and the L-lactic acid production were determined.

### Batch fermentation

Batch fermentation was conducted in a 3-L Erlenmeyer flask containing 1 L fresh medium at 50°C under static conditions. The corn stover hydrolyzate (162.5 g L^-1^) containing 83.0 g L^-1^ of total reducing sugars was used. The medium contained 9.9 g L^-1^ peanut meal and 0.3 g L^-1^ neutral proteinase, and the culture pH was maintained at 5.1-6.3 by calcium carbonate present in the medium. The well mixed samples were taken periodically and the concentrations of total residual reducing sugar and L-lactic acid production were determined.

### Analytical methods

The glucose and L-lactate concentration were measured by SBA-40D biosensor analyzer (Institute of Biology, Shandong Academy of Sciences, China). The total concentration of reducing sugars was measured by SGD-IV automatic analyzer of reducing sugar (Institute of Biology, Shandong Academy of Sciences, China). The xylose concentration was determined by xylose assay kit (Nanjing Jiancheng Technology Company Ltd, China). For quantification of formatic acid and acetatic acid, an Aminex HPX-87H column (Bio-Rad, Hercules, CA) was used. The column was maintained at 65°C and eluted with 5 mM H_2_SO_4_ at a flow rate of 0.6 mL/min. Peaks were detected by Refractive Index Detector and quantified by comparison to retention times of authentic standards. The optical purity of L-lactic acid was determined by HPLC equipped with a chiral column (MCI GEL CRS10W, Japan) at 254 nm. The mobile phase was 2 mM CuSO_4_ at a flow rate of 0.5 ml/min (25°C).
